# GMFCS and spino-pelvi-femoral complex in ambulating or walking cerebral palsy children. retrospective study

**DOI:** 10.1186/1748-7161-9-S1-O52

**Published:** 2014-12-04

**Authors:** Jean-Claude Bernard, Julie Deceuninck, Emmanuelle Chaleat-Valayer, Jessica Sutton, Sophie Leroy-Coudeville, Edith Morel, Eric Loustalet, Audrey Combey, Eric Berthonnaud

**Affiliations:** 1 CMCR des Massues, Lyon, France; 2 Hopital Nord Ouest, Villefranche sur Saone, France

## Introduction

We have performed a radiological evaluation of static data of spine-pelvis-femur complex in walking children with cerebral palsy (CP). The data are discussed about GMFCS and after about radiological data in asymptomatic subjects.

## Materials and methods

The CP population is comprised of 119 children and the asymptomatic population of 652 children.

## Results

There is no significative difference concerning the form parameter (pelvic incidence=PI), on the other hand there is a significative difference on position parameters (pelvic tilt=PT and sacral slope=SS).There is a correlation between GMFCS and PI (p=0.013) and between GMFCS and PT (p=0.021).

## Discussion

The PC population is not structurally different than the asymptomatic population. It will be the growth, in pathologic context, which disturb parameters. A lumbar lordosis which is not correlated with PI have to be consider like a result of the disease (postural troubles, neuro-motor disorders related with growth,…) and require a specific and early evaluation and treatment.

